# Diffusion Pattern and Hotspot Detection of Dengue in Belo Horizonte, Minas Gerais, Brazil

**DOI:** 10.1155/2012/760951

**Published:** 2012-03-12

**Authors:** José Eduardo Marques Pessanha Pessanha, Waleska Teixeira Caiaffa, Maria Cristina de Mattos Almeida, Silvana Tecles Brandão, Fernando Augusto Proietti

**Affiliations:** ^1^Belo Horizonte Observatory for Urban Health (OSUBH), UFMG, 30130 Belo Horizonte, MG, Brazil; ^2^Municipal Health Secretariat of Belo Horizonte, 30130 Belo Horizonte, MG, Brazil

## Abstract

This study considers the dengue occurrence in the city of Belo Horizonte over the last fifteen years. Approximately 186,000 cases registered from 1996 to 2011 were analyzed. The home address of individuals whose dengue case was notified was used as a proxy for exposure location. For determining possible outbreaks of disease and the specific patterns of dengue cases, spatial statistics used included Kernel's estimation. The occurrence of waves of dengue outbreaks was correlated with climatic and vector presence data. Outbreaks had different durations and intensities: case clustering, thinned out both spatially and temporally. These findings may be useful for public health professionals responsible for fighting the disease providing some tools for improving evaluation of interventions such as vector control and patient care, minimizing the collective and individual burden of the disease.

## 1. Introduction

The most important arbovirus disease in humans, dengue, annually affects 80 million individuals in many countries, leading to 550,000 hospitalizations and 20,000 thousand deaths [1]. The main vector is the mosquito *Aedes aegypti*, an arthropod with an extremely high capacity to adapt to urban areas.

Since 1982, the reemergence of dengue has been reported in urban centers in all Brazilian regions. The magnitude of this disease has led to high public federal, state, and municipal investments in vector control, epidemiological surveillance, and patient care.

During the 1990s, the incidence of dengue increased greatly as a consequence of the dissemination of *A. aegypti.* Dispersion of the vector was followed by the dissemination of dengue virus serotypes 1 and 2 in twenty of the 27 states of the country. Between 1990 and 2000, several epidemics occurred, mainly in the largest urban areas of the Southeast and the Northeast, where the majority of notified cases were concentrated. The first great dengue epidemic occurred in 1998, with approximately 528,000 cases [2].

In Brazil, the increase in the incidence of dengue cases in 2002 and the emergence of a third serotype (DENV-3) led to a prediction of an increased risk of dengue epidemics and an increase of the cases of dengue hemorrhagic fever (DHF). To face the expected risks for 2002, the Brazil Ministry of Health, in collaboration with the Pan-American Health Organization, carried out an international seminar in June 2000 to evaluate the dengue epidemic and to prepare a National Dengue Control Program (PNCD). However, the current epidemiological situation shows that these program measures have not achieved the expected results. Epidemiological impact assessments of these interventions have shown that their effectiveness has been extremely limited [3]. Regardless of each local health system, even when these measures are well managed, their effectiveness is always low, given the intense viral circulation detected in the successive epidemics and the results of serological surveys conducted in several Brazilian cities [4, 5].

The first dengue epidemic in Belo Horizonte (BH), the principal city of Brazil's third metropolitan area, occurred in 1996, and since then, epidemics have occurred every year. Different from the subsequent epidemics, the 1996 epidemic started in the southern hemisphere's fall. The only serotype initially identified was DENV-1. However, by the end of 1997, another epidemic of great intensity started, characterized by the simultaneous circulation of DENV-1 and DENV-2. The two serotypes continued to produce successive epidemics every year. In February 2002, DENV-3 was identified for the first time in BH, and now the three serotypes coexist [5].

The control measures, adopted in BH until the 1998 epidemic, had only a limited role, without much impact on the final numbers of cases [6]. This situation was repeated in 1997 and only changed its stance in 1998, before the largest epidemic in the city when DENV-1 and DENV-2 virus serotypes were both circulating. In 2002, it was observed that the spread of serotype 3 from the state where it was originally detected presented a different pattern from that observed with serotypes 1 and 2. Previously, the expansion of the new serotype (DENV-3) occurred slowly and some years elapsed before autochthonous cases occurred in other states. During the first three months of 2002, the presence of the new serotype was detected in ten other states. In BH, the DENV-3 serotype was isolated in only a few samples that year. It would be theoretically possible to attribute these results to the control measures proposed by the Brazil Ministry of Health in 1996, the Program of Eradication of *A. aegypti*—known as PEAa—which was only implemented in the municipality in 1998. This program took into account the difficulties of the previous control strategy and proposed an even more complex objective, predicated on the assumption that the vector could be eradicated [7].

When compared to other large urban areas in Southeast Brazil, the dengue epidemic cycle in BH has had its own characteristics [8]. Low epidemic intensity was observed from 1999 to 2005. This epidemic behavior was probably only interrupted when the resistance to the larvicidal agent being used was detected in BH in 2006 [9].

Currently, vector control is the only way to interrupt disease transmission, given that there is neither an effective vaccine nor specific therapy [10]. Vector control, however, is not a simple task, especially given the complexities of urban settings. The failure of dengue control programs has been pointed out by several authors [7, 11–13].

Spatial analyses are powerful tools in public health diagnosis and surveillance, allowing the identification of critical areas for intervention and the variables associated with the modulation of disease dynamics [14, 15]. Dengue, whose pattern is well known to be clustered in certain areas, is a health-related event for which spatial analysis techniques may be useful [16]. Spatial analyses and statistics, such as spatial autocorrelation analysis, cluster analysis, and temporal analysis, are commonly used to highlight spatial patterns of dengue cases and to test whether there is a pattern of dengue incidence in a particular area [17, 18].

A geographic information system (GIS) can be used to identify and assess potential compositional and contextual risk factors associated to disease transmission such as socioeconomic, climatic, demographic, and physical environment. GIS technologies have been applied in epidemiologic and public health studies for many years [19, 20], providing information useful for studying and modeling the spatial-temporal dynamics of dengue [21–23]. This paper aims to evaluate dengue dissemination in space and time, determining possible outbreak waves of dengue cases correlated with climatic data and presence of the vector. This study may contribute to implement interventions aimed at vector control and patient care, minimizing the collective and individual burden of this disease.

## 2. Materials and Methods

### 2.1. Study Area

**Figure 1:**
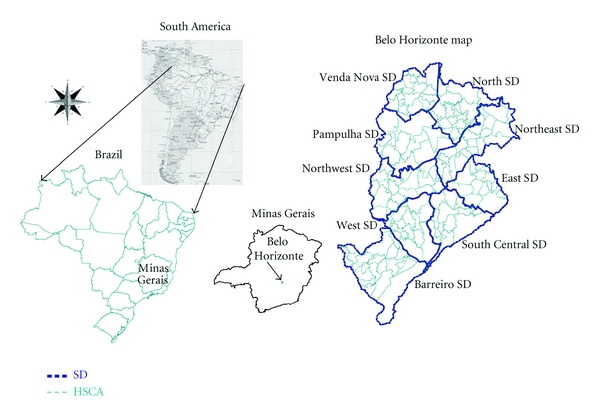
Belo Horizonte map.

This ecological study was conducted in Belo Horizonte (BH), the capital of the state of Minas Gerais, in the Southeast region of Brazil (19°55′S 43°57′W). Occupying an area of 330.23 km^2^ [24] with 2,375,151 inhabitants in approximately 600,000 households [25] (Figure 1), BH is Brazil's sixth most populous city. Situated at altitudes ranging from 700 to 1,200 meters (mean 858 meters), BH has a tropical wet and dry climate with an average annual temperature of approximately 21°C [26]. 

Each one of 147 primary care units is responsible for a geographic area known as a health services catchment area (HSCA). The HSCAs are aggregated in nine Sanitary Districts (SDs) named as North, Northeast, Northwest, East, South Central, West, Venda Nova, Pampulha, and Barreiro [27].

### 2.2. Dengue Cases

All dengue cases reported from 1996 to 2011 (partial) to the municipal surveillance system—which in turn are forwarded to Brazil's national reporting system [28]—were used. The notification form contains, along with other information, each patient's address and the date of onset of dengue symptoms.

### 2.3. Dengue Vectors

Dengue larvae vectors foci data reported for years 1996 to 2011 (partial) and eggs collected in ovitraps from 2003 to 2010 were used in this study. The data was obtained from the municipality vector reporting system—SCZOO [29] which contains the address for each larva focus and ovitrap and the dates of the survey.

The ovitraps—which cover a radius of 200 meters—are installed every two weeks [30]. The building larval index (BLI) as proposed by Connor and Monroe [31] measures the density of *A. aegypti* in urban areas and is estimated as the proportion of houses with *A. aegypti* larvae. It has been used in Brazil since 2003 and in BH since 2004.

### 2.4. Climatic Data

Rainfall (mm) and temperature (degrees Celsius) for the years 2001–2010 were obtained from weather station of the 5th district of Brazil's Meteorological Institute (INMET).

### 2.5. Spatial and Temporal Diffusion Pattern

Depending on the analysis (see below), dengue incidence was calculated on a monthly or annual basis from 1996 to 2011.

Initially, monthly temporal trends of dengue incidence were determined. Then the dengue incidence in a given year for each Sanitary District from 2005 to 2011 was correlated to September-October vector data (the mean number of eggs in the ovitraps of each SD and the BLI in the larvae foci survey) from the previous year. We used the Pearson correlation coefficient to estimate the correlation between the monthly incidence of dengue and climate data for the years 2001 to 2010.

### 2.6. Spatial Analysis

All reported cases of dengue were georeferenced using the patient household address. The vector data was geocoded using the address of the larvae foci building and the locations of the ovitraps. Spatial statistical techniques used in this study included Kernel's estimation in order to determine the possible outbreaks of disease and specific patterns of distribution on the urban space.

### 2.7. Space and Time Analysis

To find how dengue spread in space and time, we created map objects that change status with time [32].

### 2.8. Hotspot Detection

A “Hotspot” is defined as a condition indicating some form of clustering in a spatial distribution [33]. Hotspot detection can be useful, even if the global pattern is not clustered. Moreover, cases clusters that occur randomly can also have an influence on the spread of an infectious disease.

### 2.9. Software

TabWin 3.5 was used to make Brazil municipalities maps (http://www.datasus.gov.br/), and R (R Development Core Team; http://www.r-project.org/) was used to calculate the Pearson correlations and Kernel's estimation. MAPIINFO 8.5 was used to make BH hotspots maps, and Excel 2003 was used to generate tables and figures.

## 3. Results

### 3.1. Spatial and Temporal Analysis of Dengue

#### 3.1.1. Temporal Analysis

**Figure 2:**
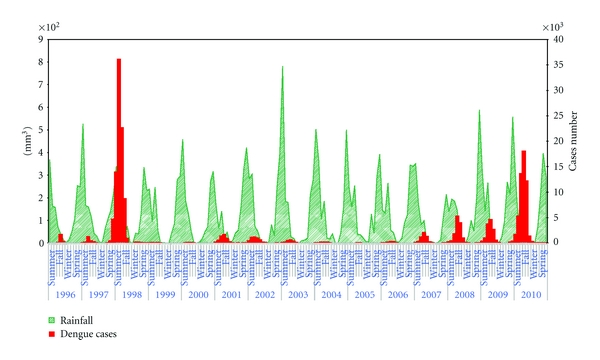
Dengue cases and rainfall by month, season, and year, Belo Horizonte, Minas Gerais state, Brazil, from 1996 to 2010.

In this series of annual incident dengue cases, five distinct periods were identified: (1) between April 1996, the first epidemic in BH, and July 1998, the most important epidemic; (2) between August 1998 and December 2000 with incidence rates not exceeding 10 cases per 100,000 inhabitants; (3) between January 2001 and August 2002, during which two new epidemics occurred; (4) between August 2002 and December 2005 again with low dengue incidence rates; (5) the last period, between January 2006 and August 2010, during which the incidence rate was progressively higher (Figure 2).

The dengue temporal distribution with highest incidence in the rainy season presented a similar pattern during the period (Figure 2). Characteristically, dengue outbreaks generally occurred during the second part of the rainy season, when humidity was higher than average [27].

In the period from 2005 to 2011, annual incidence rates of dengue showed a statistically significant correlation with the BLI according to Sanitary District (*r* = 0.60, *P* = 0.0000002). For the mean values of eggs captured in the ovitraps, the correlation was also statistically significant (*r* = 0.69, *P* = 0.00000005) (Table 1).

Rainfall (RF) and temperature (TEMP) begin to increase in October, with dengue outbreaks occurring during the months of January to May, the period of highest rainfall and humidity. The number of cases then fall through June, a period when RF and TEMP also decrease (Figure 2).

Analyzing the climatic data for the years 2001 to 2010, monthly dengue incidence rates showed a statistically significant correlation with the RF of the previous month (*r* = 0.36, *P* = 0.00006) and the monthly minimum temperature (*r* = 0.29, *P* = 0.001).

#### 3.1.2. Dengue Hotspot Detection

**Figure 3:**
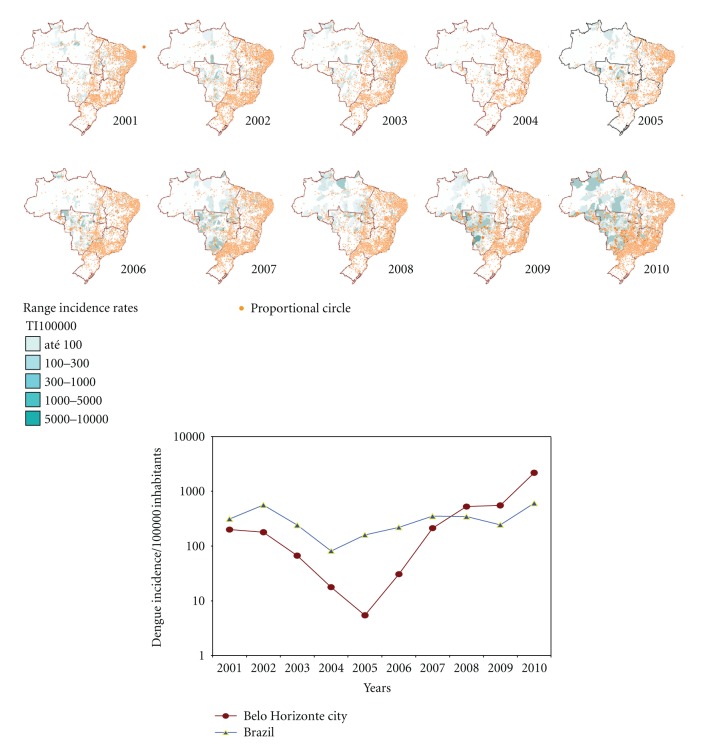
Dengue incidence point maps by city (proportional circle) and range incidence rates by municipalities areas, temporal dynamics in space, Brazil, and incidence years comparative graphic, Belo Horizonte city, Brazil, 2001–2010.

**Figure 4:**
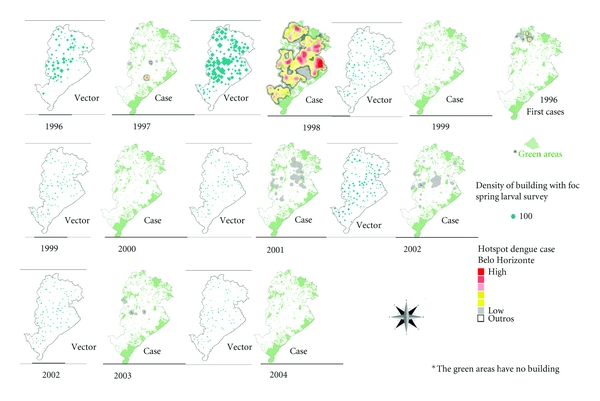
Temporal dynamics in space, foci of *Aedes aegypti *larvae in October survey and dengue outbreaks, between 1996 and 2004, Belo Horizonte/MG, Brazil.

**Figure 5:**
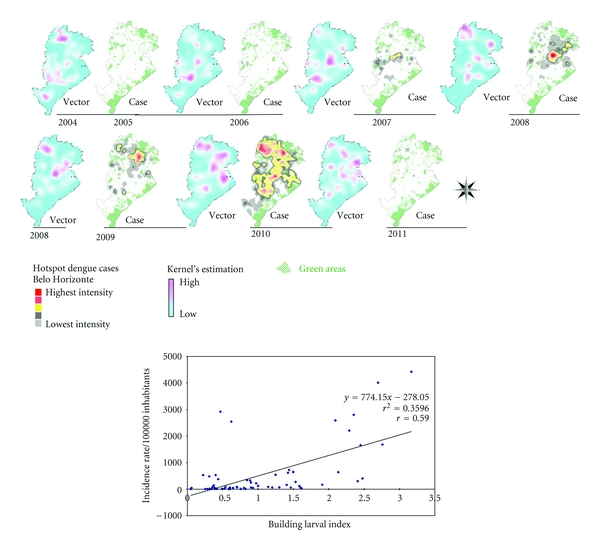
Temporal dynamics in space, foci of *Aedes aegypti *larvae Kernel's estimation in October survey and dengue hotspot outbreaks, 2004–2011, and regression linear graphic (incidence versus building larvae index), Belo Horizonte/MG, Brazil.

**Figure 6:**
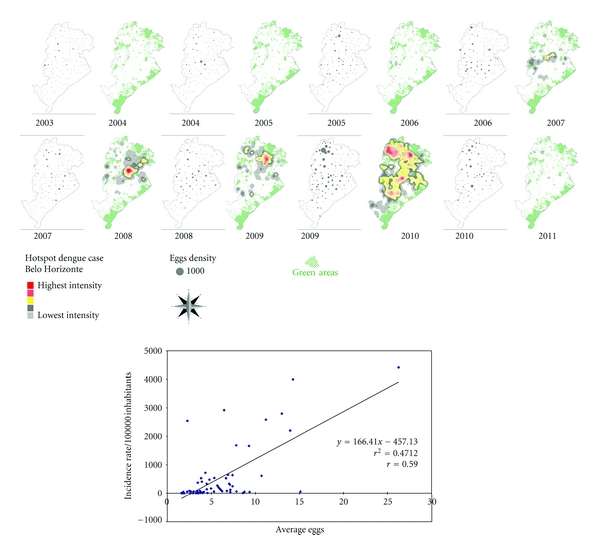
*Aedes aegypti* eggs in ovitraps, September-October survey, and dengue outbreaks, temporal dynamics in space, 2003–2011, and linear regression graphic (incidence versus mean number of eggs), Belo Horizonte, MG, Brazil.

**Figure 7:**
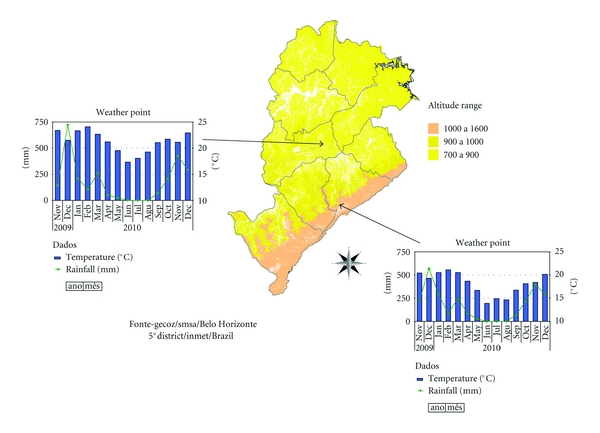
Monthly minimum temperature and rainfall, from Nov/2009 to Nov/2010, weathers geographic points, and altitude range, Belo Horizonte/MG, Brazil.

**Figure 8:**
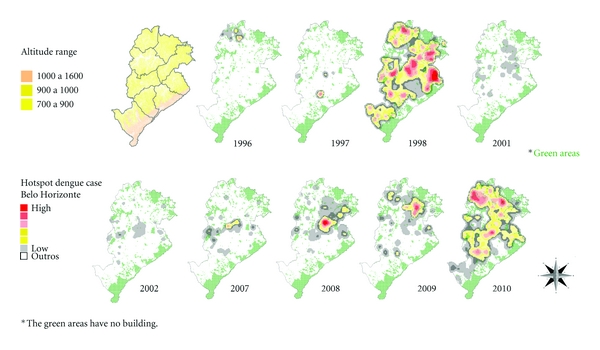
Dengue outbreaks, temporal dynamics in space, 1996–2011, and altitude range, Belo Horizonte/MG, Brazil.

The maps that comprise Figure 3 illustrate the spatial and temporal evolution of dengue in cities of Brazil and are accompanied by a comparative graph of annual incidence rates from 2001 to 2011 for BH, Brazil. Figures 4 and 5 demonstrate the spatial correlation between dengue cases hotspots and the location of *Aedes aegypti *larvae foci in BH.  Figure 6 shows the same observation among dengue cases hotspots and the areas with the greatest presence of *Aedes aegypti* eggs. The hotspot analysis also found a higher risk of dengue in areas of the city that are at lower elevations (Figures 7 and 8).

## 4. Discussion

Monitoring and planning control measures for dengue epidemics are vital for preventing or minimizing disease outbreaks. Information based on notified cases only, however, is insufficient, because many people who are infected may either be asymptomatic or do not become part of the official statistics even if they present symptoms [34].

The use of information on dengue incidences rates, mapping their patterns and dynamics of spread using spatial autocorrelation analysis, can be a valuable tool to analyze the spatial patterns change over time. Therefore, instead of aiming to achieve a complete understanding of the transmission process, it may be more efficient to improve the surveillance system and optimize disease control.

The heterogeneous intraurban distribution of dengue incidence according to Sanitary Districts for the years 2001 to 2011 suggests the importance of analyzing transmission at the SD level.

The degree of acquired immunity to the dengue virus may vary across different areas of the municipality based on the spatial distribution of previous outbreaks. Thus, Sanitary Districts with larger proportions of susceptible individuals may present higher incidences.

Our results indicate that continuous vector surveillance using ovitraps and larvae foci is necessary, so that a greater number of areas with potential transmission can be identified, permitting the prioritization and scheduling of vector control measures.

Certainly, the identification of high-risk areas, in a process of surveillance and control of the disease and the mosquito, is an important step towards optimizing resources. Once such areas have been identified, interventions may provide better results in decreasing incidences rather than through the traditional approach of a uniform control strategy for the city as a whole.

Determining whether greater vector presence or coefficients of dengue incidence predominant in certain intraurban areas may be operationalized through the use of the concept of persistence. For each SD, the number of months of uninterrupted vector presence would be calculated, thereby determining whether greater persistence occurs in specific SDs over the various periods of the year.

Temporal analysis of climatic factors (rainfall, temperature, and humidity) revealed that dengue generally occurs when average temperatures increase, when the rainy season has started, and when the humidity is higher. Previously, a report from BH showed that rainfall and relative humidity data from fifteen days before (*t*−1) showed very high correlation with dengue vector incidence in time *t* [30]. There are other studies in the literature reporting an important correlation between climate and dengue occurrences or dengue vector abundance [35–37]. However, the occurrence of a residual vector population or the occurrence of dengue cases in distinct intraurban areas in the cold and dry months, with much lower dengue incidence than in January to May, should be taken into account for disease control.

Early detection and prediction of dengue outbreaks should be goals for municipal surveillance systems. Identifying locations and patterns of the vector population (species, density, and vector-control indices) should also be used to direct interventions with disease reduction as the preferred outcome measure demonstrating impact, and ovitraps index, house index, container index, and Breteau index as proxy indicators of impact. With these strategies, information will be available in real time, which may uncover other aspects about the relationship between vector and the disease that could be revealed through spatial analyses [38, 39].

Other tools such as the industrial control chart—proposed by Rich and Terry [40], and adopted in several survey vigilance systems—when applied to dengue require several improvements related to presentation and interpretation in order to enhance its usefulness. The ability to demonstrate trends, analyzing only notified dengue cases at a potentially earlier time point, is limited. Heterogeneous internet access limits the use of query-based surveillance web tools to identify disease and location outbreaks as candidates for interventions. Although this proposal is intriguing, so far the identification of a given outbreak is usually too late for control measures.

## 5. Conclusions

Our findings show that the strategies used in this study can help public health officials to visualize and understand the geographic distribution and trends of disease patterns and to prepare warnings and awareness campaigns. Dengue spatial and temporal spread patterns and hotspot detection may constitute useful information for public health officials to control and predict dengue dissemination from critical hotspot areas. This may save time and cost and make public health department actions more efficient. Public health officers may employ the model to plan a strategy to control dengue by analyzing the information received on distribution and hotspots for various months. Some ancillary findings of the study such as influence of climate, which is seasonal and thus temporal, also contribute to knowledge regarding its significance. The methodology is based on principles of spatial statistics and has the potential to be applied to other epidemics. In the future, it will be important to have regular daily statistics accumulated over several years to permit faster recognition of outbreak locations and be prepared to promptly implement appropriate public health interventions.

## Figures and Tables

**Table 1:** Building larval index (BLI) in October larval foci survey, eggs average (EA) in September-October survey, and dengue incidence rates (DIR) per 100,000 inhabitants in subsequent years, by Sanitary District, Belo Horizonte city, 2004–2010.

Sanitary District											
Year		Barreiro	South central	East	Northeast	Northwest	North	West	Pampulha	Venda nova	BH
2004	Building larval index	0.4	0.3	0.0	0.3	0.3	0.3	0.3	0.3	0.6	0.3
	Eggs (average)	1.9	1.6	1.7	1.9	3.5	2.6	3.6	2.9	3.6	2.6
	Dengue incidence rate	1.1	2.6	2.0	5.5	5.6	2.1	2.2	3.5	2.9	3.2

2005	Building larval index	0.5	0.8	0.4	0.5	0.5	0.6	0.9	0.6	0.8	0.6
	Eggs (average)	3.2	4.0	3.3	3.8	3.7	4.6	7.1	4.1	8.6	4.7
	Dengue incidence rate	7.6	6.0	5.6	17.9	81.5	5.7	44.3	46.3	5.7	26.1

2006	Building larval index	1.6	1.5	1.4	2.5	2.1	1.1	1.9	2.4	1.6	1.8
	Eggs (average)	3.9	5.3	5.9	3.9	7.4	2.5	5.0	7.1	6.1	5.2
	Dengue incidence rate	29.4	58.3	160.2	403.2	637.3	83.1	167.1	299.4	114.5	232.4

2007	Building larval index	0.1	0.4	0.4	0.6	0.3	0.2	0.4	0.4	1.0	0.4
	Eggs (average)	1.8	4.1	3.6	2.4	4.8	5.9	4.6	3.8	5.7	4.0
	Dengue incidence rate	45.0	71.8	369.7	2,545.8	477.5	529.0	139.2	528.7	217.9	558.6

2008	Building larval index	0.4	0.7	0.9	1.2	0.9	0.5	0.8	1.4	1.5	0.9
	Eggs (average)	2.4	2.8	4.4	5.4	8.3	6.3	7.0	4.2	6.6	5.2
	Dengue incidence rate	53.0	62.8	329.6	540.8	245.1	2,922.6	336.1	719.5	646.9	563.4

2009	Building larval index	1.4	1.5	2.8	2.4	2.1	2.7	2.3	2.4	3.2	2.3
	Eggs (average)	11.0	6.1	7.7	9.8	11.8	14.1	14.2	12.9	26.2	12.3
	Dengue incidence rate	678.1	273.3	1,837.1	1,823.3	2,511.9	4,371.3	2,316.0	3,802.2	5,111.5	2,375.3

2010	Building larval index	0.6	0.6	1.3	1.2	0.7	1.0	0.8	1.6	0.9	0.9
	Eggs (average)	2.7	3.8	6.2	7.8	6.8	7.2	9.3	8.7	15.1	7.3
	Dengue incidence rate	61.2	35.3	69.4	61.8	79.7	118.3	43.0	49.6	56.8	63.3

*Pearson correlation BLI and DIR: r = 0.59, P < 0.001.*

*Pearson correlation EA and DIR: r = 0.69, P < 0.001.*
